# Prediction of novel target genes and pathways involved in irinotecan-resistant colorectal cancer

**DOI:** 10.1371/journal.pone.0180616

**Published:** 2017-07-27

**Authors:** Precious Takondwa Makondi, Chi-Ming Chu, Po-Li Wei, Yu-Jia Chang

**Affiliations:** 1 International Ph.D. Program in Medicine, College of Medicine, Taipei Medical University, Taipei, Taiwan, ROC; 2 Graduate Institute of Clinical Medicine, College of Medicine, Taipei Medical University, Taipei, Taiwan, ROC; 3 School of Public Health, National Defense Medical Center, Taipei, Taiwan, ROC; 4 Department of Surgery, College of Medicine, Taipei Medical University, Taipei, Taiwan, ROC; 5 Division of Colorectal Surgery, Department of Surgery, Taipei Medical University Hospital, Taipei Medical University, Taipei, Taiwan; 6 Cancer Research Center, Taipei Medical University Hospital, Taipei, Taiwan, ROC; 7 Translational Laboratory, Department of Medical Research, Taipei Medical University Hospital, Taipei Medical University, Taipei, Taiwan; 8 Graduate Institute of Cancer Biology and Drug Discovery, Taipei Medical University, Taipei, Taiwan; Columbia University, UNITED STATES

## Abstract

**Background:**

Acquired drug resistance to the chemotherapeutic drug irinotecan (the active metabolite of which is SN-38) is one of the significant obstacles in the treatment of advanced colorectal cancer (CRC). The molecular mechanism or targets mediating irinotecan resistance are still unclear. It is urgent to find the irinotecan response biomarkers to improve CRC patients’ therapy.

**Methods:**

Genetic Omnibus Database GSE42387 which contained the gene expression profiles of parental and irinotecan-resistant HCT-116 cell lines was used. Differentially expressed genes (DEGs) between parental and irinotecan-resistant cells, protein-protein interactions (PPIs), gene ontologies (GOs) and pathway analysis were performed to identify the overall biological changes. The most common DEGs in the PPIs, GOs and pathways were identified and were validated clinically by their ability to predict overall survival and disease free survival. The gene-gene expression correlation and gene-resistance correlation was also evaluated in CRC patients using The Cancer Genomic Atlas data (TCGA).

**Results:**

The 135 DEGs were identified of which 36 were upregulated and 99 were down regulated. After mapping the PPI networks, the GOs and the pathways, nine genes (GNAS, PRKACB, MECOM, PLA2G4C, BMP6, BDNF, DLG4, FGF2 and FGF9) were found to be commonly enriched. Signal transduction was the most significant GO and MAPK pathway was the most significant pathway. The five genes (FGF2, FGF9, PRKACB, MECOM and PLA2G4C) in the MAPK pathway were all contained in the signal transduction and the levels of those genes were upregulated. The FGF2, FGF9 and MECOM expression were highly associated with CRC patients’ survival rate but not PRKACB and PLA2G4C. In addition, FGF9 was also associated with irinotecan resistance and poor disease free survival. FGF2, FGF9 and PRKACB were positively correlated with each other while MECOM correlated positively with FGF9 and PLA2G4C, and correlated negatively with FGF2 and PRKACB after doing gene-gene expression correlation.

**Conclusion:**

Targeting the MAPK signal transduction pathway through the targeting of the FGF2, FGF9, MECOM, PLA2G4C and PRKACB might increase tumor responsiveness to irinotecan treatment.

## Introduction

Colorectal cancer (CRC) is the third most frequently diagnosed cancer and the third leading cause of cancer deaths worldwide representing 10% of the world-wide cancer incidence and mortality[1]. Surgical removal of the tumor is the first choice treatment for non-metastatic CRC though approximately one-quarter of CRC patients have metastases at diagnosis and half developing metastases even after complete resection[2,3]. The addition of cytotoxic drugs oxaliplatin and irinotecan and the monoclonal anti-bodies cetuximab, bevacizumab or panitunumab to the backbone of the antimetabolite 5-fluorouracil has improved the median survival of metastatic CRC(mCRC) from 8 to 24 months[4–6]. Drug resistance still hampers the efficient treatment of mCRC as half of the patients have intrinsic or acquired resistance contributing to a 5-year survival rate of 60% to 65%[7–10] and with the chemotherapy side effects being many, there is an unmet need for therapy response predictive biomarkers[11].

Attempts to identify chemotherapy predictive biomarkers of treatment response and resistance has yielded some results with high thymidylate synthase (TS) expression being a predictor of poorer outcome in 5-fu-based therapy and also 5-fu adjuvant treatment being ineffective in tumors with microsatellite instability[12–14];likewise higher levels of TOP1 is correlated with greater sensitivity of colon tumors to camptothecin derivatives compared with normal colonic mucosa, but there is no irinotecan predictive biomarkers that have reached a level of evidence allowing for routine clinical use[15].

The testing of predictive biomarkers is not applied routinely in clinical practice, also in prediction studies on response of colon cancer cells, it has been demonstrated that the assessment of multiple biomarkers provides accurate prediction of drug response than a single biomarker[16–18]. Gene expression profiling has been applied effectively in classifying CRC molecular tumor subtypes and several studies have shown the feasibility of identifying genes involved in the progression and the prognosis of CRC[16,19]. However the mechanism of acquired resistance to irinotecan are not fully understood[20,21].

This study used microarray gene expression profile to identify biomarkers and pathways involved in the acquired resistance to irinotecan in CRC and we tested these biomarkers in the prediction of irinotecan resistance, overall survival and disease free survival in clinical data of patients with metastatic colon cancer.

## Methods and materials

### Microarray data

The Gene Expression Omnibus (GEO) database (http://www.ncbi.nlm.nih.gov/geo/) gene expression profile with accession number GSE42387 was downloaded. The data was sequenced on the platform of GPL16297 Agilent-014850 Whole Human Genome Microarray 4x44K G4112F (Agilent Systematic Name, collapsed probe, version). The GSE42387 dataset had 27 samples which included in triplicate three parental human colon cancer cell lines of HCT116, HT29 and LoVo and their acquired resistant subset generated after being exposed in vitro to gradually increasing concentrations of oxaliplatin or irinotecan for nine months. MTT colorimetric assays were used to determine the 50% inhibitory concentration (IC_50_) for oxaliplatin or irinotecan resistant and the parental cell lines with the sensitivity criteria of IC_50_ of <1 μM for Oxaliplatin and <1nM for irinotecan. Then the total RNA was extracted and converted into cDNA through reverse transcription and transcribed with T7 RNA Polymerase. Then, the labeled cRNA was hybridized to Agilent Human Gene Expression Microarrays and scanned using an Agilent DNA Microarray scanner. The quality of the RNA was assessed using a Bio analyzer 2100 and microarray data was processed in R (www.r-project.org) using the Bioconductor bioinformatics software package (http://www.bioconductor.org/) using the Limma package [22,23]and normalized between arrays using quantile normalization[24] and the expression values were log2 transformed.

### Data preprocessing and DEGs screening

The GEO2R online analytical tool[25], which uses the R language in applying GEOquery and Limma packages was used to recalculate gene expressions. The HCT116 parental cell lines and their corresponding irinotecan resistant cell lines were selected to identify the differentially expressed genes (DEGs) between parental and resistant cell lines. The t-test method was utilized to calculate the p-values of genes. Then, Benjamin & Hochberg's method [26]was used to calculate the adjusted p-values (false discovery rate, FDR) the DEGs with the log2 fold change (FC) of > 1 or < - 1 and FDR <0.05 were selected.

### Hierarchical clustering analysis

After extracting the expression values from the gene expression profile, a bidirectional hierarchical clustering heatmap was constructed using multiExperimental Viewer(MEV) v4.8 software[27].

### Construction of PPI network

In the construction of the PPI networks, STRING version 10.0 (http://www.string-db.org/) [28]was used. This is a web biological database for prediction of known and unknown protein interaction relationships. A combined score of >0.7(High confidence) was selected as as the cut-off criterion. Then, the PPI pairs were inputted into Cytoscape software version 3.4.0 (http://www.cytoscape.org)[29] to construct the PPI network. The highly connected proteins (hub nodes) with important biological functions were identified by calculating the number of lines connecting the proteins (the degree) and how much nodes that are not directly connected by a certain node (betweenness value) of each node by CytoNCA app for cytoscape with the node degree cutoff criterion of ≥2[30].

### Enrichment analysis of DEGs

The database for Annotation, Visualization and Integrated Discovery (DAVID, https://david.ncifcrf.gov) [31]was used to classify significant DEGs by their biological processes, molecular functions, or cellular components using Gene Ontology consortium reference(GO, http://www.geneontology.org/) [32]and the significant transcripts(Benjamini-Hochberg FDR <0.05) were identified using the David Functional Annotation clustering tool[31]. The DAVID database was also used to perform pathway enrichment analysis with reference from Kyoto Encyclopedia of Genes and Genomes (KEGG, http://www.genome.jp/kegg/) database website and Benjamini-Hochberg FDR <0.05 as a cut-off point[33,34].

### Clinical validation of the DEGs

For clinical assessment of the DEGs identified to be associated with irinotecan resistance, *survExpress*[35], an online biomarker validation tool was used to perform survival analysis. The colon metabase including GSE12945, GSE14333, GSE17536, GSE17537, GSE31595 and GSE41258 with a total of 808 cases was chosen. The survival profiles were compared based on high or low mRNA expression of a particular gene and assessed independently for overall survival and disease free survival in months. The hazard ratio(HR) with 95% confidence intervals(CI) and log rank P-value (<0.05 as significance value) were calculated and the results were downloaded and loaded in SPSS version 21 for plotting of Kaplan-Meier survival curves.

### Confirmation of irinotecan resistant genes

To evaluate the candidate genes’ role in predicting irinotecan resistance, RNA expression profiles generated from TCGA-COAD RNA-sequence dataset (level 3.1.12.0) were used. The patients were divided into two groups: those who received irinotecan and had recurrent tumors within one-year treatment, were defined as irinotecan resistant. In contrast, the patients who did not develop tumor relapse after one-year of irinotecan treatment were classified as irinotecan sensitive (responders). There were nine irinotecan-resistant and five irinotecan- sensitive patients in the TCGA-COAD RNA sequence dataset. The gene expression level in this dataset was represented in log2-transformed RSEM format. The resistant genes were found by comparing the expression level of each target gene in the irinotecan-sensitive and resistant patients and the significance value was found by the Mann-Whitney U test method with <0.05 as significant.

### Gene co-expression in colorectal cancer data

The cancer genome atlas (TCGA; https://cancergenome.nih.gov/) was used to find colo- rectal cancer data which contained gene expression profiles. Level 3 RNA-Seq data containing 635 colorectal cancer cases with gene expression profiles (463 colon adenocarcinoma cases and 172 rectal adenocarcinoma cases) was downloaded, Standard Pearson correlation coefficients (-1 to 1) of the desired gene pairs were calculated using SPSS version 21 software with significance level p-value of <0.05 was set as the cutoff criteria.

## Results

### DEGs in GEO2R

**Fig 1:**
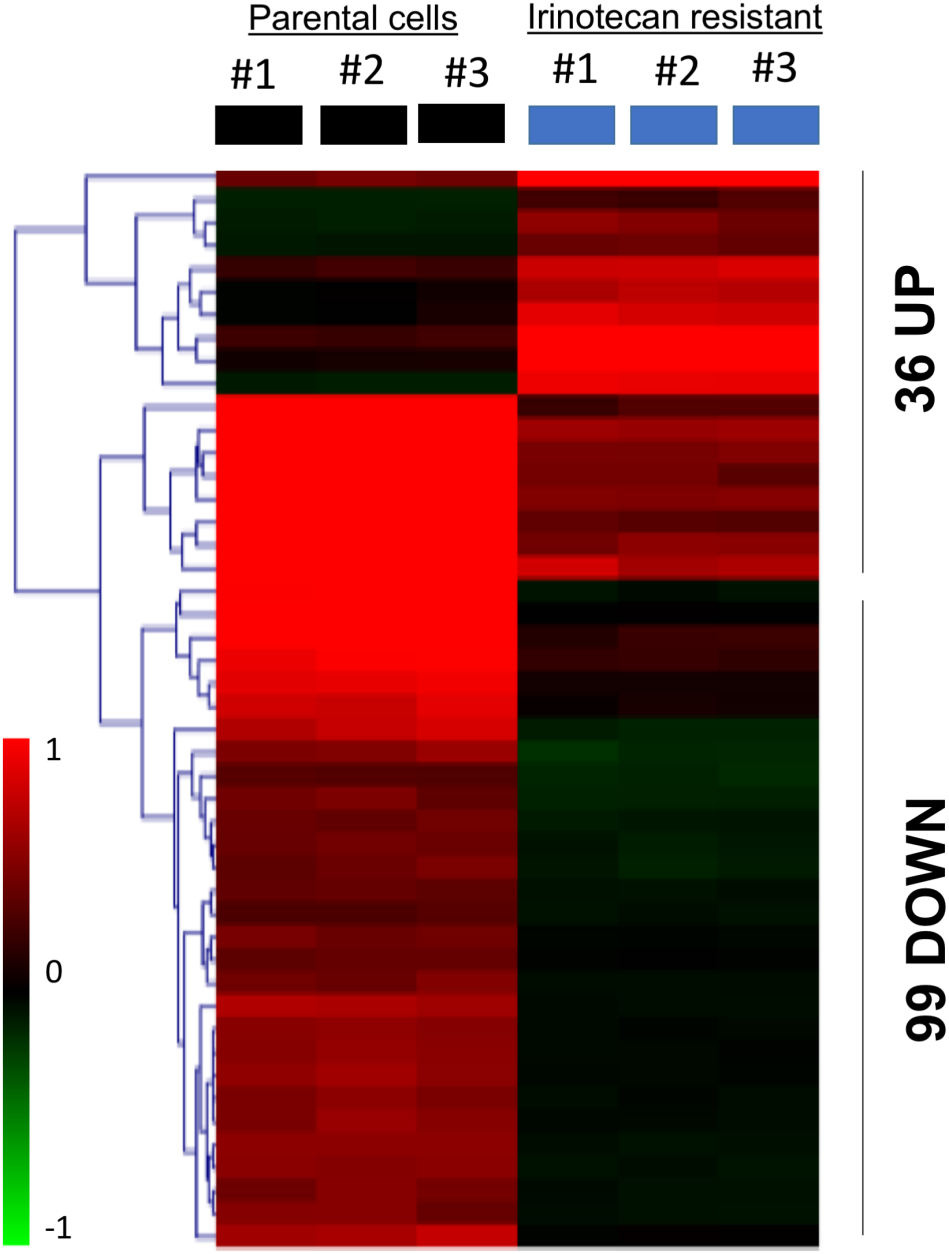
Heat map showing upregulated and downregulated differentially expressed genes (DEGs) in irinotecan-resistant colon cancer cells. A bidirectional hierarchical clustering heat map Constructed using multiExperimental Viewer(MEV). The expression values are log2 fold changes (>1 or <−1, FDR <0.05)) between corresponding irinotecan-resistant HCT116 cell lines and parental HCT116 cell lines. Black represents no change in expression, green represents downregulation, and red represents upregulation.

The data derived from the GPL16297 oligonucleotide microarray platform by using the GEOR2 tool consisted of 32,701 probe sets. Then 135 DEGs were identified to be related to irinotecan resistance after calculation of log2FC and FDR values, of which 36 were upregulated and 99 were downregulated, then gene expression values were extracted and a bidirectional hierarchical clustering heat map was plotted using the the MultiExperiment Viewer v4.8 software to present the DEGs (Fig 1, S1 and S2 Tables).

### PPI networks

**Fig 2:**
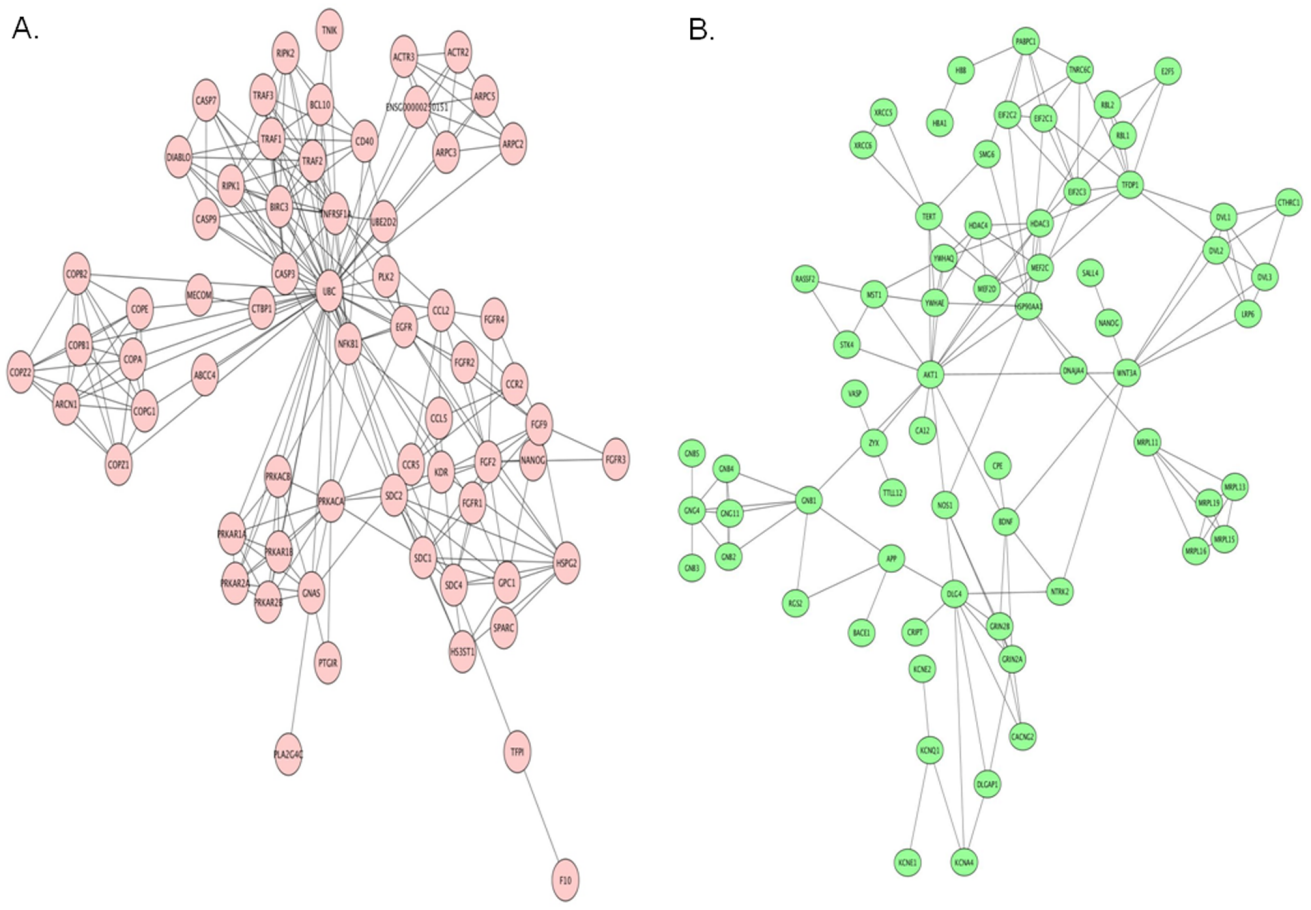
Protein–protein interaction (PPI) network of differentially expressed genes (A) upregulated genes and (B) downregulated genes. The PPI pairs were imported into Cytoscape software as described in Methods and Materials. Pink nodes represent up regulated genes while green nodes represent down regulated genes. The lines represent interaction relationship between nodes.

After mapping gene and protein interactions using STRING database to obtain the PPI pairs, the PPI pairs were imported into Cytoscape software. The upregulated DEGs network contained 83 nodes and 238 edges, 7 DEGs; PRKACB, GNAS, FGF2, FGF9, HS3ST1, PLA2G4C and MECOM had higher degrees and betweenness values (Fig 2A, Table 1). In the down regulated group which contained 147 nodes and 149 edges, 7 DEGs; BDNF, WNT3A, BMP6, HDAC4, DLG4, CAMK1 and CPE had higher degrees and betweenness values(Fig 2B, Table 2).

**Table 1:** Up-regulated genes which had interactions in the protein–protein interaction (PPI) network.

Gene symbol	Gene name	Degree	Betweenness
PRKACB	protein kinase, cAMP-dependent, catalytic, beta	34.0	237.86037
GNAS	GNAS complex locus	28.0	56.964874
FGF2	fibroblast growth factor 2 (basic)	28.0	289.1067
FGF9	fibroblast growth factor 9 (glia-activating factor)	19.0	52.558647
HS3ST1	heparan sulfate (glucosamine) 3-O-sulfotransferase 1	7.0	0.0
PLA2G4C	phospholipase A2, group IVC (cytosolic, calcium-independent)	4.0	0.4871795
MECOM	MDS1 And EVI1 Complex Locus	2.0	0.0

**Table 2:** Down-regulated genes which had interactions in the protein–protein interaction (PPI) network.

Gene symbol	Gene name	Degree	Betweenness
BDNF	brain-derived neurotrophic factor	28.0	39.905716
WNT3A	wingless-type MMTV integration site family, member 3A	25.0	14.574099
HDAC4	histone deacetylase 4	16.0	6.1847615
DLG4	discs, large homolog 4 (Drosophila)	15.0	4.1179338
BMP6	bone morphogenetic protein 6	13.0	3.048329
CAMK1	calcium/calmodulin-dependent protein kinase I	8.0	0.79026806
CPE	carboxypeptidase E	4.0	0.2857143

#### GO analysis

DAVID online tool was used to classify the 135 DEGs according to their common GO functions of biological processes, molecular functions, or cellular components. We included 1454 GO gene sets in the reference database, of which 37 were significantly enriched (with p < 0.05) and had an FDR of <0.05. The most significant gene set was signal transduction (GO:0007165) (FDR = 6.9E-1, p = 1.3E-3) which contained 18 genes. The top 10 GOs are summarised in the Table 3.

**Table 3:** The top ten enriched gene ontologies (GOs).

GO term	Count	FDR	P-value
Signal transduction(GO:0007165)	18	6.9E-1	1.3E-3
cellular response to glucagon stimulus(GO:0071377)	4	6.4E-1	2.2E-3
positive regulation of osteoblast differentiation (GO:0045669)	4	8.8E-1	7.0E-3
positive regulation of protein binding (GO:0032092)	4	8.1E-1	7.3E-3
axon guidance(GO:0007411)	5	9.8E-1	2.0E-2
Inner ear development(GO:0048839)	3	9.9E-1	2.9E-2
bone development(GO:0060348)	3	9.8E-1	3.0E-2
heart development(GO:0007507)	5	9.7E-1	3.1E-2
Phosphatidylinositol-3-phosphate biosynthetic process(GO:0036092)	3	9.9E-1	4.0E-2
positive regulation of canonical Wnt signaling pathway(GO:0090263)	4	9.8E-1	4.3E-2

GO, Gene ontology; FDR, False discovery rate

### KEGG pathway analysis

**Fig 3:**
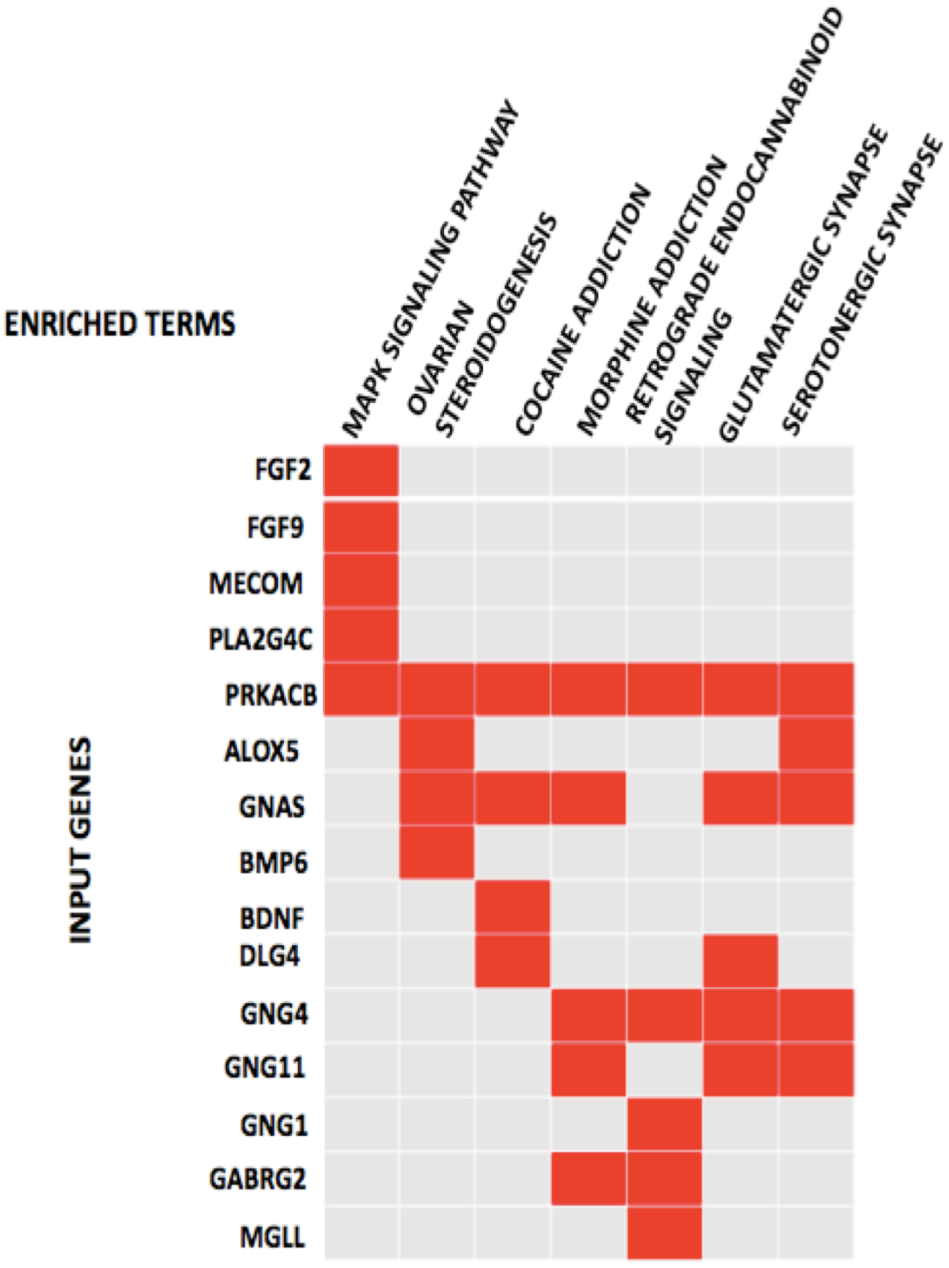
Significant KEGG pathways and the genes involved. Gene enrichment analysis of the DEGs involved in irinotecan resistance showing KEGG pathways significantly enriched in irinotecan resistant cell lines and the genes involved in the pathways (the pathways in order of their enrichment from left to right) (FDR <0.05 p-value of <0.05).

**Fig 4:**
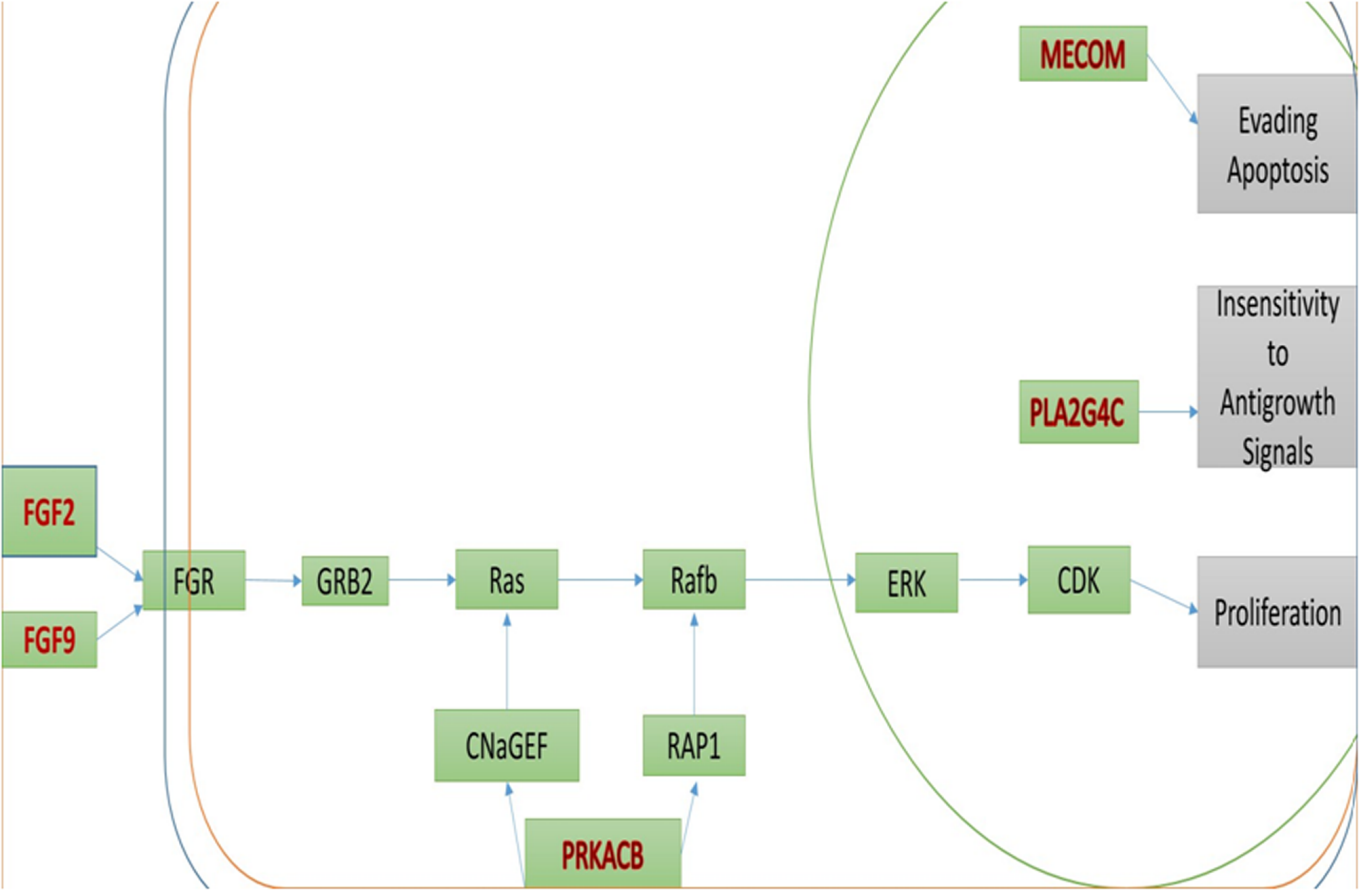
Simplified MAPK pathway showing the location of the significant genes in the pathway. The DAVID online tool was used to download the pathway and show the position of the DEGs in the pathway using the pathway ID of hsa04010. Input genes are in red. And the final effect of the pathway is represented in gray.

DAVID online tool was also used to classify the 135 DEGs using the KEGG pathway reference. The KEGG pathway analysis indicated that 7 pathways reached the statistical significance (p-value <0.05, FDR <0.05) including the MAPK signaling pathway, ovarian steroidogenesis, cocaine addiction, morphine addiction, retrograde endocannabinoid signaling, glutamatergic synapse and serotonergic synapse (Fig 3 and Table 4). Next the MAPK signaling pathway ID of hsa04010 was used to locate the position of the enriched genes in the pathway using DAVID online tool, the pathway was summarized and presented in Fig 4.

**Table 4:** Enriched KEGG pathways.

KEGG pathway	Count	FDR	P-value
MAPK signaling pathway (hsa04010)	5	0.0025	0.0001
ovarian steroidogenesis (hsa04913)	4	0.0149	0.0003
cocaine addiction (hsa05030)	4	0.0149	0.0003
morphine addiction (hsa05032)	5	0.0149	0.0004
retrograde endocannabinoid signaling (hsa04723)	5	0.0181	0.0006
glutamatergic synapse (hsa04724)	5	0.0208	0.0010
serotonergic synapse (hsa04726)	5	0.0208	0.0009

KEGG, Kyoto Encyclopedia of Genes and Genomes; FDR, False discovery rate

### Common genes between PPI networks, GOs and pathway analysis

Nine genes (GNAS, PRKACB, MECOM, PLA2G4C, BMP6, BDNF, DLG4, FGF2 and FGF9) were observed to be commonly enriched after the analysis of the PPI networks, GOs and pathways. GNAS, PRKACB, MECOM, PLA2G4C, FGF2 and FGF9 were up-regulated and BMP6, BDNF, DLG4 were down-regulated. To further compare with both the signal transduction and the MAPK pathway, we found that five genes were significantly enriched including PRKACB, MECOM, PLA2G4C, FGF2 and FGF(Tables 3 and 4). It was also observed that all these genes were upregulated (Fig 3).

### Genes which correlate to colorectal cancer patient survival

**Fig 5:**
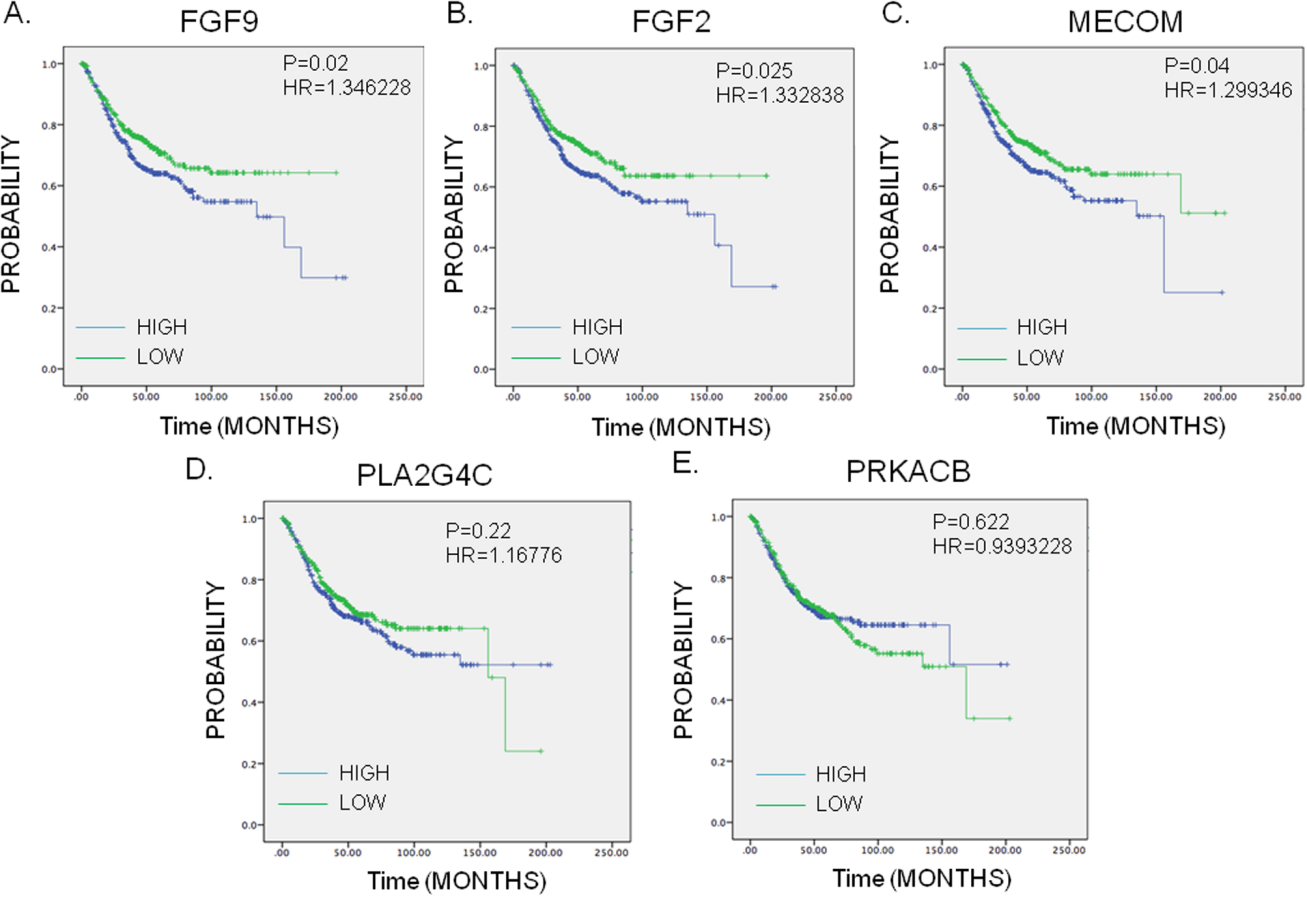
Kaplan-Meier survival curves presenting the prognostic relationship between high and low expression of specific genes involved in irinotecan resistance to overall survival (A) MECOM, (B) FGF2, (C) FGF9, (D) PLA2G4C and (E) PRKACB. The survival curves were plotted using the *survExpress* online tool. The specific DEGs expression levels were dichotomized by median value and the results presented visually by Kaplan-Meier survival plots. P-values were calculated using log-rank statistics. Patient number = 808, HR = Hazard Ratio, P = Logrank P-value.

To further check the role of PRKACB, MECOM, PLA2G4C, FGF2 and FGF in colorectal cancer, we further analyzed the genes involved in the MAPK pathway for their association with patient’s survival using the *survExpress* online tool. As shown in Fig 5, three genes; MECOM, FGF2 and FGF9 were associated with survival with high expression of the genes correlating with poor survival (Fig 5A–5C) while PLA2G4C and PRKACB did not predict survival (Fig 5C and 5D). The combination of 5 genes showed the highly association with patients’ outcome (S1 Fig). In addition, we also analyzed the correlation of those five genes with disease free survival. We found highly FGF9 expression was associated with poor disease free survival with all the other 4 genes not significant (S2A–S2E Fig).

### Mechanism of gene correlation in tumor tissues

**Fig 6:**
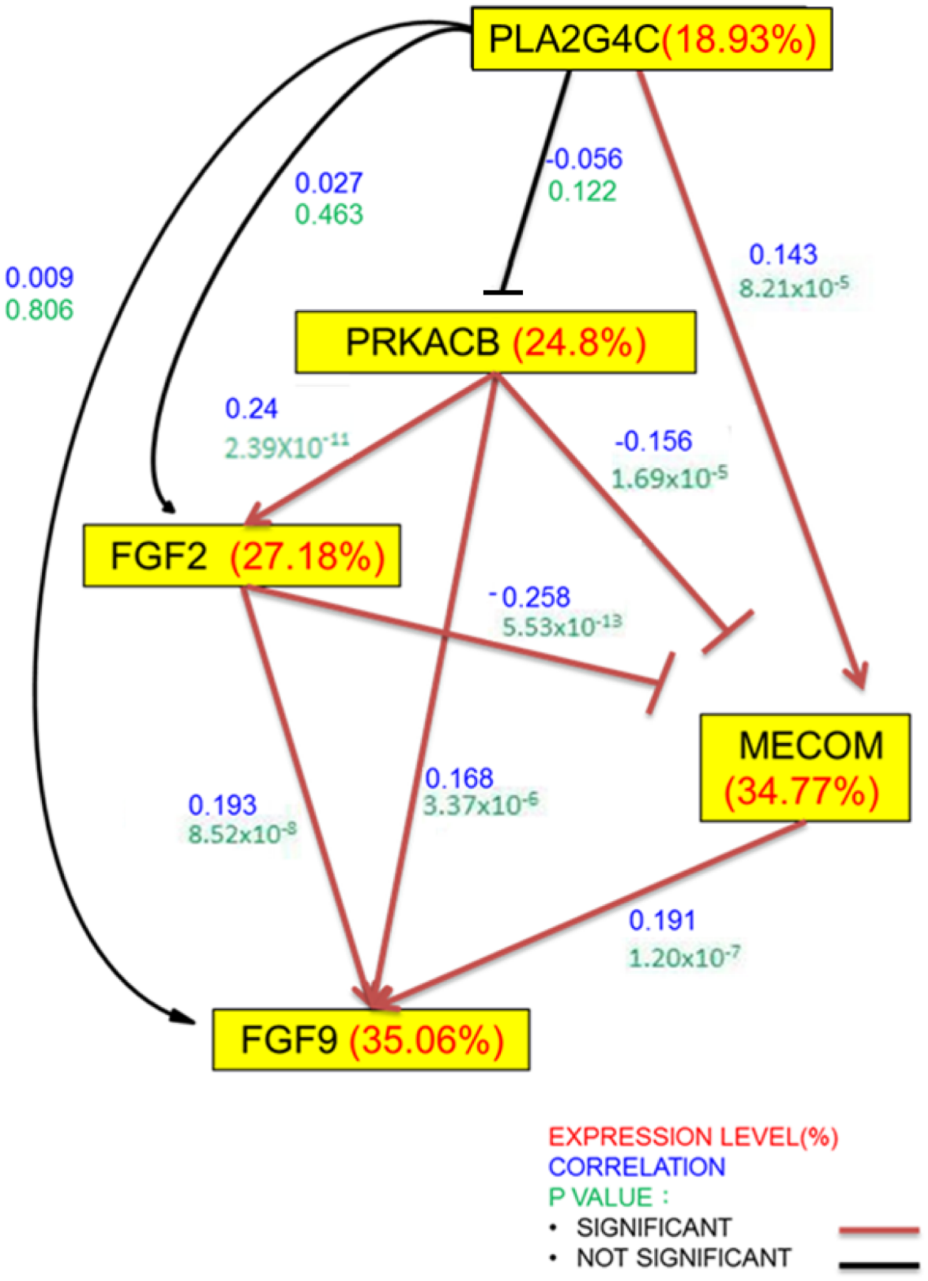
Gene expression correlation of the genes involved in the MAPK pathway in the CRC tumor samples from the TCGA data base. The pooled microarray datasets downloaded from NCBI were used to identify the relationship of these candidate genes. The genes in the upstream of the network were assumed to be less variant, so coefficient of variation (CV) of each gene (presented in percent) was calculated to determine its site in the regulatory network. Besides, Spearman’s rank correlation coefficients (-1 to 1) of the desired gene pairs decide whether they were negative/positive control with significance level p<0.05. Number of patients = 653. The red lines or arrows represent significant correlation (P-value <0.05). Coefficient of variation in red, correlation coefficient in blue and P-value in green.

To understand the mechanism of gene-gene correlation expression of PRKACB, MECOM, PLA2G4C, FGF2 and FGF, the pooled microarray datasets downloaded from NCBI were used to identify the relationship of these candidate genes. As shown in Fig 6, we found that FGF2, FGF9 and PRKACB were positively correlated with each other. MECOM correlated positively with FGF9 and PLA2G4C, but correlated negatively with FGF2 and PRKACB (Fig 6)

### Genes which correlated to irinotecan resistance colorectal cancer patients

**Fig 7:**
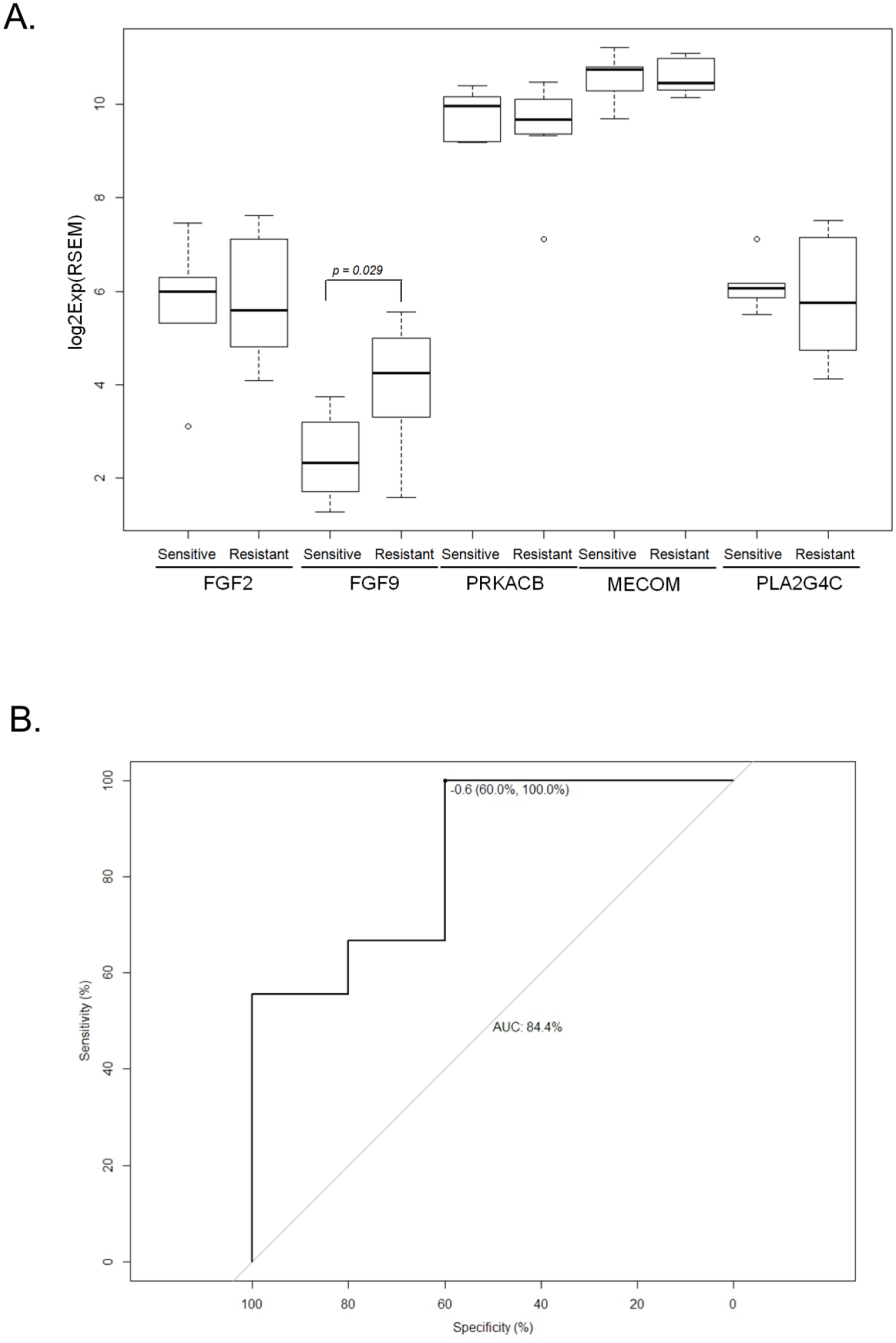
**(A)Box plots presenting the gene expression status between irinotecan sensitive and irinotecan resistant patients of the five candidate genes (MECOM, FGF2, FGF9, PLA2G4C and PRKACB). (B) The receiver operating characteristic (ROC) curve showing area under ROC curve (AUC) and the threshold of score between FGF9 and PLA2G4C**. The regression model was also estimated based on the two genes and the score was calculated representing irinotecan resistance. TCGA-COAD RNA-sequence dataset (level 3.1.12.0) was used. The patients were divided into sensitive and resistant. The significance value was found by the Mann-Whitney U test method (P <0.05 as significant). Number of patients = 14.

The data generated from TCGA-COAD RNA-sequence dataset comprising 14 irinotecan treated patients of which nine were resistant and five were responders, was used. To evaluate the roles of the candidate genes in irinotecan resistance, box plots were plotted comparing gene expression between the sensitive and the resistant group. It was found that only FGF9 was significantly elevated in irinotecan-resistant patients as compared to irinotecan sensitive patients (p = 0.029, Mann-Whitney U test). The other genes had no significant difference between the irinotecan-sensitive and resistant groups (Fig 7A).

To further evaluate the explanation power of each candidate gene, logistic regression was run and the significance was tested. The results showed that only FGF9 and PLA2C4G significantly contributed to irinotecan resistance. The regression model was estimated based on the two genes and the score was calculated representing irinotecan resistance. This model was followed by receiver operating characteristic (ROC) analysis[36]. Its area under the ROC curve (AUC) was found to be 84.4%. The threshold of score, calculated by Youden’s method, was found to be -0.6 with 100% sensitivity and 60% specificity (Fig 7B).

## Discussion

CRC remains a significant cause of morbidity and mortality worldwide with high disease incidence and significant numbers of patients presenting with advanced, metastatic disease[37]. Despite advances in medical and surgical therapy, the 5-year overall survival rate is still is low at 60% to 65% [10]. The development of resistance to irinotecan treatment is still the major challenge in managing CRC [38]. The mechanisms leading to development of irinotecan-resistance are poorly characterized. The understanding of the etiological factors and mechanisms of irinotecan-resistance will help to improve the therapeutic efficacy of CRC patients.

In the present study, we demonstrated that FGF9, FGF2, MECOM, PRKACB and PLA2G4C are the new candidate genes for irinotecan resistance. To further check the correlation between those five genes with overall survival or disease free survival rate, FGF9, FGF2 and MECOM were associated with survival rate and only FGF9 was associated with disease free survival (Fig 5, S2 Fig). The possible explanation for the inconsistency may be that this analysis is based on the general CRC patients who were not specifically receiving irinotecan therapy. This may be needed to be verified by specific irinotecan treatment cohort.

Our finding has revealed that FGF9 is positively correlated with FGF2, PRKACB and MECOM therefore placing it at a central role (Fig 6). All these genes have been correlated to resistance status in different cancers [39–44], though the role of those genes in irinotecan resistance is still unclear. In addition, FGF9 is also the only gene that predicted irinotecan resistance and disease free survival making it an important gene in that, the targeting of FGF9 might affect the expression levels and prognostic and therapeutic effects of FGF2, PRKACB and MECOM. The effects of FGF9 were also observed with its interaction with PLA2G4C in the irinotecan resistant cohort (Fig 7B). Thus FGF9 was found to play a crucial role as it was involved in all the stages in this study and was also observed to interact with all other genes.

FGFs interact with FGF receptors for signal transduction which regulates cell growth and differentiation and have also been implicated in tumorigenesis and induction of drug resistance in cancer cells [45–47]. The targeting of either FGF2/FG2R or FGF9 has been shown to increase the sensitivity of chemotherapy [39,48–52]. Those studies also support our finding that the upregulation of FGF2 and FGF9 appears to reduce the response to irinotecan.

Besides FGFs, we also found that MECOM is related to irinotecan resistance in CRC and also has a prognostic role. MECOM is a nuclear zinc finger transcription factor [53] involved in the cell cycle, proliferation, and differentiation and is said to suppress the JNK1-mediated phosphoration of c-Jun thereby exhibiting antiapoptotic effects[54]. MECOM is a transcription factor with no clear downstream targets and with no transcription profile been reported before, therefore more in-depth research on the relationship of FGF9 and MECOM biological functions could lead to the discovery of potential irinotecan targets, which can hasten a new chemical drug therapy for metastatic CRC which will improve the survival time and life quality of CRC patients therefore increasing the significance of FGF9 and MECOM in irinotecan treatment in CRC.

The other two identified biomarkers play a role either in resistance or in prognosis in cancer. PRKACB may involve in type 1 cAMP-dependent protein kinase a-related pathway which modulates breast, pancreatic, colon and ovarian cancers to be resistant to different cytotoxic agents [55–57]. PLA2G4C plays the prognostic role in breast and colon cancer [44,58].

MAPK signal transduction pathway restrains the efficacy of irinotecan in CRC. The modifications in the MAPK signal transduction pathway increase resistance to irinotecan with the upregulation of growth factors responsible for tumor growth CRC[59]. The MAPK p38 which is required for cell proliferation and survival, is also said to be activated in irinotecan resistant HCT116 CRC cells[60–62]. The pharmacological inhibition of MAPK p38 may overcome irinotecan resistance both in vitro and in vivo by mediating cell cycle arrest and autophagy-mediated cell death[60–62].

The findings provide important new target genes which could predict irinotecan treatment response and the emergence of resistance. This approach can also predict patient prognosis however further studies need to be conducted to validate these findings and determine whether they can be applied in a clinical setting.

## Conclusion

In conclusion, targeting the MAPK signal transduction pathway through the targeting of FGF9, FGF2, MECOM, PRKACB and PLA2G4C might increase tumor responsiveness to irinotecan and improve patient survival thus being therapeutically and prognostic significant in CRC patients.

## Supporting information

S1 Fig Kaplan-Meier survival curves presenting the overall prognostic relationship between high and low expression of target genes (MECOM, FGF2, FGF9, PLA2G4C and PRKACB) involved in irinotecan resistance to overall survival. The survival curves were plotted using the *survExpress* online tool. The target genes’ expression levels were dichotomized by median value and the results presented visually by Kaplan-Meier survival plots. P-values were calculated using log-rank statistics. Patient number = 808, HR = Hazard Ratio, P = Logrank P-value.
(TIF)

S2 Fig Kaplan-Meier survival curves presenting the relationship between high and low expression of specific genes to disease free survival (A) FGF9, (B) FGF2, (C) MECOM, (D) PLA2G4C and (E) PRKACB expression. The survival curves were plotted using the *survExpress* online tool. The genes’ expression levels were dichotomized by median value and the results presented visually by Kaplan-Meier survival plots. P-values were calculated using log-rank statistics. Patient number = 808, HR = Hazard Ratio, P = Logrank P-value.
(TIF)

S1 Table Up-regulated differentially expressed irinotecan-resistant genes. (DOCX)

S2 Table Down-regulated differentially expressed irinotecan-resistant genes. (DOCX)
