# Supplementary figures and images for: Prediction of novel target genes and pathways involved in irinotecan-resistant colorectal cancer
Source: PLoS One. 2017 Jul 27;12(7):e0180616. doi: 10.1371/journal.pone.0180616 (PMC5531462; doi:10.1371/journal.pone.0180616)

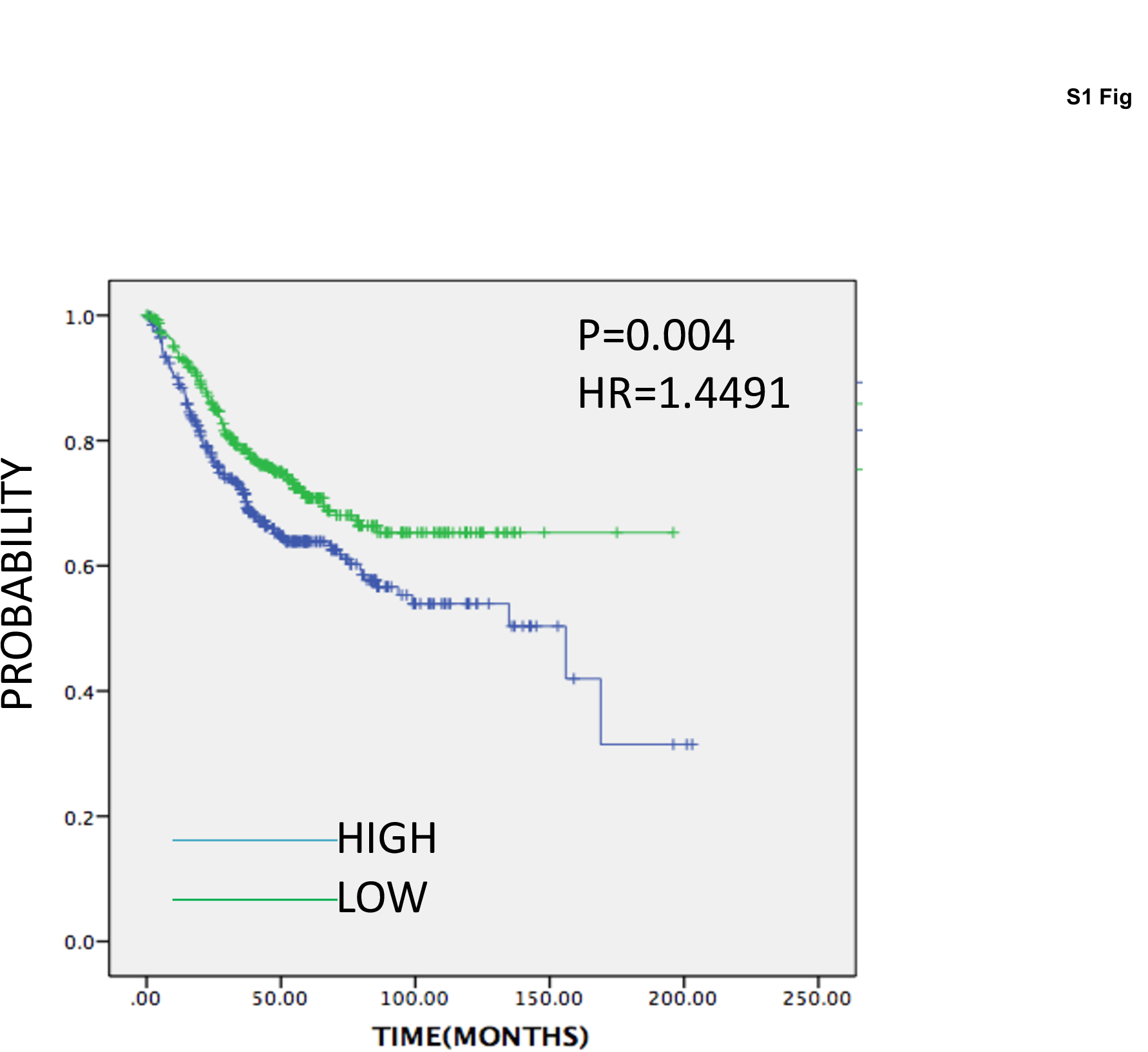

Supplement: S1 Fig — The survival curves were plotted using the survExpress online tool. The target genes’ expression levels were dichotomized by median value and the results presented visually by Kaplan-Meier survival plots. P-values were calculated using log-rank statistics. Patient number = 808, HR = Hazard Ratio, P = Logrank P-value. (TIF) [file pone.0180616.s001.tif]

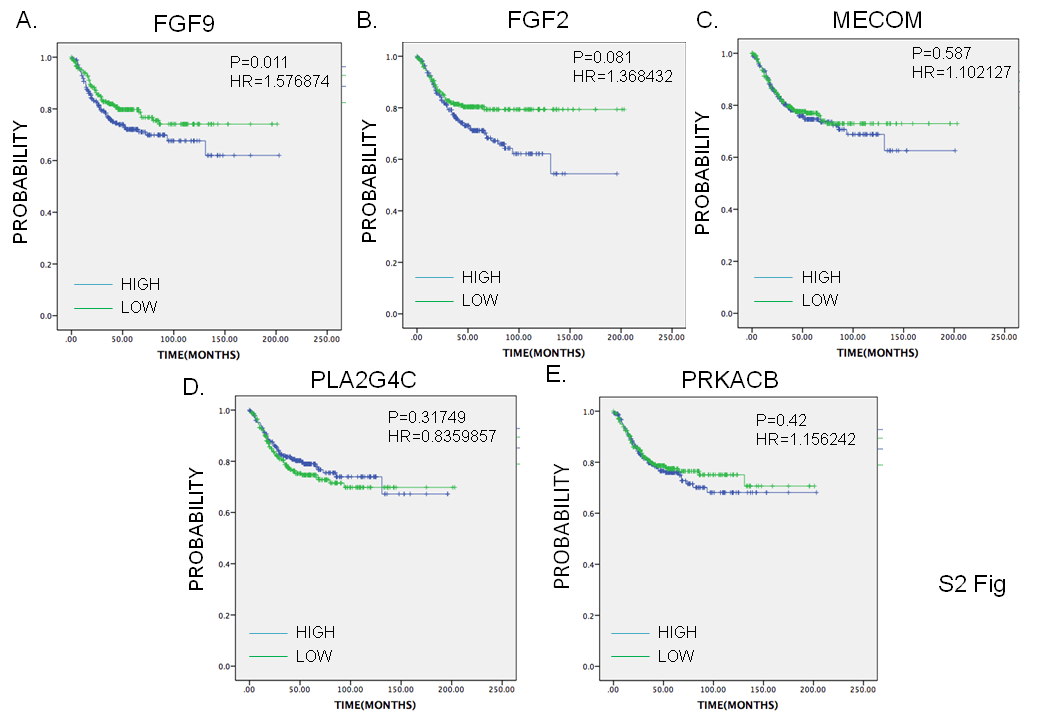

Supplement: S2 Fig — The survival curves were plotted using the survExpress online tool. The genes’ expression levels were dichotomized by median value and the results presented visually by Kaplan-Meier survival plots. P-values were calculated using log-rank statistics. Patient number = 808, HR = Hazard Ratio, P = Logrank P-value. (TIF) [file pone.0180616.s002.tif]
