# Supplementary material for: Prediction of novel target genes and pathways involved in irinotecan-resistant colorectal cancer
Source: PLoS One. 2017 Jul 27;12(7):e0180616. doi: 10.1371/journal.pone.0180616 (PMC5531462; doi:10.1371/journal.pone.0180616)
Supplement: S1 Table — (DOCX) [file pone.0180616.s003.docx]

**S1 Table : Up-regulated genes**

| **Gene ID** | **Log FC** | **FDR** | **P-value** |
| --- | --- | --- | --- |
| \| ALDH1L1 \| \| --- \| \| SPARC \| \| ABCC4 \| \| LOC730755 \| \| SFTA1P \| \| ALOX5 \| \| LOC387895 \| \| GLIS3 \| \| PRSS23 \| \| INPP4B \| \| ARPC4-TTLL3 \| \| ITGBL1 \| \| BIRC3 \| \| FGF9 \| \| GNAS \| \| C10orf140 \| \| LOC100507460 \| \| SLC2A14 \| \| PLA2G4C \| \| HS3ST1 \| \| ANO2 \| \| ZDHHC11 \| \| SULF2 \| \| TFPI \| \| MGLL \| \| PRKACB \| \| PLK2 \| \| TNIK \| \| KRT32 \| \| FGF2 \| \| COPB2 \| \| CAPS2 \| \| CCL2 \| \| MECOM \| \| CDH15 \| | \| 3.476 \| \| --- \| \| 3.324 \| \| 2.946 \| \| 2.782 \| \| 2.444 \| \| 2.403 \| \| 1.844 \| \| 1.71 \| \| 1.706 \| \| 1.625 \| \| 1.602 \| \| 1.602 \| \| 1.562 \| \| 1.551 \| \| 1.522 \| \| 1.498 \| \| 1.45 \| \| 1.409 \| \| 1.401 \| \| 1.393 \| \| 1.391 \| \| 1.31 \| \| 1.309 \| \| 1.305 \| \| 1.287 \| \| 1.275 \| \| 1.265 \| \| 1.261 \| \| 1.205 \| \| 1.185 \| \| 1.182 \| \| 1.111 \| \| 1.066 \| \| 1.05 \| \| 1.019 \| | \| 6.15E-07 \| \| --- \| \| 1.35E-04 \| \| 2.94E-06 \| \| 4.26E-04 \| \| 3.96E-05 \| \| 2.19E-05 \| \| 8.58E-06 \| \| 3.08E-05 \| \| 2.19E-05 \| \| 5.88E-05 \| \| 5.88E-05 \| \| 6.18E-05 \| \| 6.98E-05 \| \| 3.04E-04 \| \| 3.67E-04 \| \| 3.27E-04 \| \| 3.96E-05 \| \| 3.89E-04 \| \| 5.88E-05 \| \| 2.52E-04 \| \| 7.49E-04 \| \| 4.66E-04 \| \| 8.18E-04 \| \| 1.76E-04 \| \| 3.04E-04 \| \| 4.60E-04 \| \| 2.96E-04 \| \| 1.60E-04 \| \| 4.26E-04 \| \| 1.62E-04 \| \| 4.85E-04 \| \| 1.19E-04 \| \| 1.19E-04 \| \| 5.44E-04 \| \| 3.27E-04 \| | \| 1.88E-11 \| \| --- \| \| 3.01E-07 \| \| 5.39E-10 \| \| 1.94E-06 \| \| 4.88E-08 \| \| 2.05E-08 \| \| 4.46E-09 \| \| 3.11E-08 \| \| 2.07E-08 \| \| 9.69E-08 \| \| 9.78E-08 \| \| 1.06E-07 \| \| 1.30E-07 \| \| 9.95E-07 \| \| 1.41E-06 \| \| 1.15E-06 \| \| 5.09E-08 \| \| 1.52E-06 \| \| 9.47E-08 \| \| 7.31E-07 \| \| 5.27E-06 \| \| 2.37E-06 \| \| 6.22E-06 \| \| 4.32E-07 \| \| 9.95E-07 \| \| 2.25E-06 \| \| 9.23E-07 \| \| 3.77E-07 \| \| 1.93E-06 \| \| 3.86E-07 \| \| 2.55E-06 \| \| 2.52E-07 \| \| 2.40E-07 \| \| 2.99E-06 \| \| 1.15E-06 \| |
