# Supplementary material for: Prediction of novel target genes and pathways involved in irinotecan-resistant colorectal cancer
Source: PLoS One. 2017 Jul 27;12(7):e0180616. doi: 10.1371/journal.pone.0180616 (PMC5531462; doi:10.1371/journal.pone.0180616)
Supplement: S2 Table — (DOCX) [file pone.0180616.s004.docx]

**S2 Table: Down-regulated genes**

| \| **Gene** \|  \|  \|  \| \| --- \| --- \| --- \| --- \| | **Log FC** | **FDR** | **P-value** |
| --- | --- | --- | --- | --- | --- | --- | --- |
| \| CRIP1 \|  \| \| --- \| --- \| \| REEP2 \|  \| \| CSTA \|  \| \| GNG4 \|  \| \| WNT3A \|  \| \| KLK10 \|  \| \| GNE \|  \| \| ARMC4 \|  \| \| WWC3 \|  \| \| NETO2 \|  \| \| SQLE \|  \| \| CNTLN \|  \| \| RAB40B \|  \| \| VASP \|  \| \| TACSTD2 \|  \| \| SUSD3 \|  \| \| E2F5 \|  \| \| C10orf58 \|  \| \| CA12 \|  \| \| KCNQ1 \|  \| \| CD33 \|  \| \| ITPKB \|  \| \| TERT \|  \| \| RASL10A \|  \| \| GPAT2 \|  \| \| SALL4 \|  \| \| MKX \|  \| \| RGS2 \|  \| \| XXYLT1 \|  \| \| KIAA1257 \|  \| \| ZC3H6 \|  \| \| BACE1 \|  \| \| ZNF544 \|  \| \| MCCC2 \|  \| \| RNF135 \|  \| \| KRT86 \|  \| \| NKAIN1 \|  \| \| KRT80 \|  \| \| ADAM19 \|  \| \| STOX1 \|  \| \| CPE \|  \| \| STK39 \|  \| \| SRGAP1 \|  \| \| FAM176B \|  \| \| CRABP2 \|  \| \| KLK6 \|  \| \| GNG11 \|  \| \| SUN3 \|  \| \| TRNP1 \|  \| \| FTL \|  \| \| UCA1 \|  \| \| CTHRC1 \|  \| \| TRIOBP \|  \| \| APOC1 \|  \| \| FAM184A \|  \| \| MIPEP \|  \| \| CKMT1A \|  \| \| DLG4 \|  \| \| C2orf70 \|  \| \| BDNF \|  \| \| HBA1 \|  \| \| PKD2 \|  \| \| TNRC6C \|  \| \| IL17B \|  \| \| GABRG2 \|  \| \| MRPL15 \|  \| \| UROS \|  \| \| DNAJA4 \|  \| \| BTBD2 \|  \| \| FAM134B \|  \| \| CITED4 \|  \| \| PACSIN1 \|  \| \| C1orf106 \|  \| \| BMP6 \|  \| \| GPRC5C \|  \| \| SH3D19 \|  \| \| LOC15109 \|  \| \| DGKG \|  \| \| RASSF2 \|  \| \| TRIM15 \|  \| \| CAMK1 \|  \| \| FAR2 \|  \| \| B4GALNT \|  \| \| EHBP1L1 \|  \| \| HDAC4 \|  \| \| RAB2A \|  \| \| CHDH \|  \| \| COCH \|  \| \| CCDC122 \|  \| \| SPIRE1 \|  \| \| TTLL12 \|  \| \| UNC5A \|  \| \| TYSND1 \|  \| \| FAM116A \|  \| \| CYP2U1 \|  \| \| NSMAF \|  \| \| TCTN1 \|  \| \| ANK3 \|  \| | \| -4.349 \| \| --- \| \| -3.481 \| \| -3.318 \| \| -3.212 \| \| -2.741 \| \| -2.418 \| \| -2.416 \| \| -2.269 \| \| -2.253 \| \| -2.221 \| \| -2.208 \| \| -2.207 \| \| -2.203 \| \| -2.189 \| \| -2.161 \| \| -2.102 \| \| -2.038 \| \| -2.037 \| \| -2.017 \| \| -2.002 \| \| -1.983 \| \| -1.92 \| \| -1.894 \| \| -1.891 \| \| -1.879 \| \| -1.874 \| \| -1.858 \| \| -1.849 \| \| -1.801 \| \| -1.791 \| \| -1.763 \| \| -1.756 \| \| -1.748 \| \| -1.684 \| \| -1.626 \| \| -1.589 \| \| -1.57 \| \| -1.556 \| \| -1.544 \| \| -1.54 \| \| -1.539 \| \| -1.533 \| \| -1.513 \| \| -1.48 \| \| -1.462 \| \| -1.46 \| \| -1.433 \| \| -1.427 \| \| -1.417 \| \| -1.409 \| \| -1.397 \| \| -1.382 \| \| -1.353 \| \| -1.341 \| \| -1.337 \| \| -1.318 \| \| -1.314 \| \| -1.3 \| \| -1.288 \| \| -1.267 \| \| -1.263 \| \| -1.261 \| \| -1.243 \| \| -1.226 \| \| -1.223 \| \| -1.22 \| \| -1.21 \| \| -1.207 \| \| -1.199 \| \| -1.191 \| \| -1.185 \| \| -1.184 \| \| -1.173 \| \| -1.165 \| \| -1.152 \| \| -1.149 \| \| -1.133 \| \| -1.129 \| \| -1.118 \| \| -1.117 \| \| -1.112 \| \| -1.109 \| \| -1.099 \| \| -1.094 \| \| -1.09 \| \| -1.088 \| \| -1.074 \| \| -1.06 \| \| -1.055 \| \| -1.05 \| \| -1.037 \| \| -1.032 \| \| -1.028 \| \| -1.025 \| \| -1.021 \| \| -1.016 \| \| -1.014 \| \| -1.004 \| | \| 1.27E-06 \| \| --- \| \| 3.79E-06 \| \| 3.11E-04 \| \| 1.51E-06 \| \| 1.23E-05 \| \| 4.26E-04 \| \| 2.94E-06 \| \| 2.43E-04 \| \| 3.79E-06 \| \| 4.07E-06 \| \| 6.24E-05 \| \| 1.57E-05 \| \| 1.88E-05 \| \| 3.62E-06 \| \| 4.18E-04 \| \| 3.16E-05 \| \| 3.79E-06 \| \| 9.64E-06 \| \| 6.41E-06 \| \| 5.68E-04 \| \| 5.88E-05 \| \| 1.88E-05 \| \| 8.04E-04 \| \| 1.52E-05 \| \| 1.76E-04 \| \| 1.57E-05 \| \| 5.58E-05 \| \| 5.87E-05 \| \| 1.23E-05 \| \| 1.05E-05 \| \| 3.96E-05 \| \| 3.38E-04 \| \| 9.82E-06 \| \| 3.77E-05 \| \| 6.65E-04 \| \| 1.19E-04 \| \| 1.70E-05 \| \| 6.56E-04 \| \| 3.96E-05 \| \| 2.78E-04 \| \| 2.50E-04 \| \| 3.67E-04 \| \| 5.55E-05 \| \| 1.19E-04 \| \| 3.98E-04 \| \| 3.35E-05 \| \| 4.48E-04 \| \| 1.80E-04 \| \| 1.77E-04 \| \| 4.59E-04 \| \| 6.81E-04 \| \| 3.96E-05 \| \| 6.86E-04 \| \| 4.14E-04 \| \| 5.88E-05 \| \| 1.52E-04 \| \| 3.27E-04 \| \| 6.81E-04 \| \| 2.34E-04 \| \| 3.94E-04 \| \| 3.64E-04 \| \| 3.27E-04 \| \| 3.04E-04 \| \| 2.19E-04 \| \| 7.76E-05 \| \| 1.35E-04 \| \| 7.90E-04 \| \| 7.92E-04 \| \| 7.49E-04 \| \| 5.55E-05 \| \| 3.43E-04 \| \| 9.20E-05 \| \| 1.75E-04 \| \| 4.26E-04 \| \| 5.97E-04 \| \| 2.48E-04 \| \| 8.90E-05 \| \| 3.64E-04 \| \| 2.97E-04 \| \| 4.15E-04 \| \| 5.41E-04 \| \| 1.50E-04 \| \| 6.53E-04 \| \| 5.51E-04 \| \| 4.47E-04 \| \| 3.04E-04 \| \| 7.64E-04 \| \| 1.50E-04 \| \| 6.67E-04 \| \| 2.82E-04 \| \| 2.90E-04 \| \| 1.89E-04 \| \| 4.15E-04 \| \| 4.64E-04 \| \| 6.65E-04 \| \| 2.54E-04 \| \| 4.64E-04 \| \| 4.28E-04 \| | \| 7.79E-11 \| \| --- \| \| 1.04E-09 \| \| 1.04E-06 \| \| 1.56E-10 \| \| 7.99E-09 \| \| 1.95E-06 \| \| 5.00E-10 \| \| 6.82E-07 \| \| 1.39E-09 \| \| 1.62E-09 \| \| 1.09E-07 \| \| 1.19E-08 \| \| 1.57E-08 \| \| 7.74E-10 \| \| 1.79E-06 \| \| 3.29E-08 \| \| 1.37E-09 \| \| 5.31E-09 \| \| 3.13E-09 \| \| 3.31E-06 \| \| 9.28E-08 \| \| 1.61E-08 \| \| 6.07E-06 \| \| 1.07E-08 \| \| 4.36E-07 \| \| 1.20E-08 \| \| 8.36E-08 \| \| 8.97E-08 \| \| 8.24E-09 \| \| 6.41E-09 \| \| 4.81E-08 \| \| 1.22E-06 \| \| 5.70E-09 \| \| 4.26E-08 \| \| 4.21E-06 \| \| 2.42E-07 \| \| 1.35E-08 \| \| 4.09E-06 \| \| 4.70E-08 \| \| 8.34E-07 \| \| 7.18E-07 \| \| 1.42E-06 \| \| 8.14E-08 \| \| 2.49E-07 \| \| 1.62E-06 \| \| 3.58E-08 \| \| 2.17E-06 \| \| 4.63E-07 \| \| 4.43E-07 \| \| 2.23E-06 \| \| 4.38E-06 \| \| 4.99E-08 \| \| 4.45E-06 \| \| 1.70E-06 \| \| 9.90E-08 \| \| 3.53E-07 \| \| 1.12E-06 \| \| 4.39E-06 \| \| 6.51E-07 \| \| 1.57E-06 \| \| 1.38E-06 \| \| 1.11E-06 \| \| 9.95E-07 \| \| 6.02E-07 \| \| 1.47E-07 \| \| 2.92E-07 \| \| 5.80E-06 \| \| 5.83E-06 \| \| 5.17E-06 \| \| 8.00E-08 \| \| 1.26E-06 \| \| 1.80E-07 \| \| 4.24E-07 \| \| 1.85E-06 \| \| 3.58E-06 \| \| 7.04E-07 \| \| 1.71E-07 \| \| 1.38E-06 \| \| 9.37E-07 \| \| 1.72E-06 \| \| 2.93E-06 \| \| 3.40E-07 \| \| 4.05E-06 \| \| 3.13E-06 \| \| 2.14E-06 \| \| 9.88E-07 \| \| 5.54E-06 \| \| 3.44E-07 \| \| 4.26E-06 \| \| 8.54E-07 \| \| 8.96E-07 \| \| 5.10E-07 \| \| 1.75E-06 \| \| 2.30E-06 \| \| 4.21E-06 \| \| 7.45E-07 \| \| 2.32E-06 \| \| 2.01E-06 \| |
